# PROTOCOL: What is the impact of complex WASH interventions on gender and social equality outcomes in low‐ and middle‐income countries? A mixed‐method systematic review protocol

**DOI:** 10.1002/cl2.1164

**Published:** 2021-05-16

**Authors:** Biljana Macura, Laura Del Duca, Adriana Soto, Naomi Carrard, Louisa Gosling, Karin Hannes, James Thomas, Lewnida Sara, Marni Sommer, Hugh S. Waddington, Sarah Dickin

**Affiliations:** ^1^ Stockholm Environment Institute Stockholm Sweden; ^2^ Institute of Development Studies University of Sussex Brighton UK; ^3^ Institute for Sustainable Futures University of Technology Sydney Australia; ^4^ WaterAid London UK; ^5^ Research group SoMeTHin'K (Social, Methodological and Theoretical Innovation Kreative), CESO, Faculty of Social Sciences KU Leuven Leuven Belgium; ^6^ Department of Social Science, EPPI‐Centre UCL Institute of Education London UK; ^7^ Water, World Bank Nairobi Kenya; ^8^ Department of Sociomedical Sciences, Mailman School of Public Health Columbia University New York City New York USA; ^9^ London School of Hygiene and Tropical Medicine London UK

## 1 BACKGROUND

### 1.1 The problem: Limited evaluation of social and gender equality outcomes of water, sanitation and hygiene interventions

Safely managed water, sanitation and hygiene (WASH) services are viewed as fundamental for human wellbeing, enabling a range of positive outcomes related to health, education, livelihoods, dignity, safety, and gender equality. Progress in providing WASH services and thus achieving these outcomes has not occurred equally, with a range of inequalities in who can access and benefit from WASH services across varying socio‐cultural contexts, geographical areas and socioeconomic settings. For instance, among the 785 million people who lack a basic drinking‐water service, and 2 billion who lack access to basic sanitation services, a greater proportion are poor and living in rural areas (WHO/UNICEF JMP, 2019). Further, unsafely managed water and sanitation disproportionately impacts a number of social groups, including women, girls, and sexual and gender minorities, people with disabilities, people marginalised due to ethnicity, caste, poverty or other factors, and those living in vulnerable situations such as displaced people or people who are experiencing homelessness. As the COVID‐19 pandemic disproportionately affects particular groups of people, it has the potential to exacerbate many of these existing WASH inequalities (Howard et al., 2020).

Gender inequalities related to WASH are particularly large, as women and girls have specific needs related to biological factors, and experience strongly gendered norms surrounding water and sanitation, such as expectations of carrying out water fetching, caregiving and hygiene roles within the household (Caruso et al., 2015). In many countries where women and girls are responsible for water fetching this contributes to a substantial burden of musculoskeletal disease (Geere & Cortobius, 2017). Additionally, women and girls are more negatively impacted by a lack of private and safe sanitation facilities, particularly for menstrual hygiene management which creates sanitation‐related psychosocial stress and may cause urinary tract infections (Das et al., 2015; Torondel et al., 2018). Maternal and child health are also thought to be seriously affected by inadequate WASH—for example, sepsis, one of the biggest causes of neonatal mortality, due to unhygienic practices by mothers and birth attendants(Campbell et al., 2015). Additionally, a lack of a household toilet and the practice of open defecation has been linked to sexual violence (Jadhav et al., 2016). These inequalities extend beyond the household, with women and girls, and socially marginalised groups often under‐represented in decision‐making processes at all levels of WASH governance (Coulter et al., 2019; Shrestha & Clement, 2019). In particular, women have had limited access to skilled and higher‐paid employment in the water sector such as within water utilities (World Bank, 2019). While the WASH sector has frequently focused on women and a binary understanding of gender, sexual, and gender minorities also experience a range of WASH‐related inequalities (Boyce et al., 2018; Neves‐Silva & Martins, 2018; Schmitt et al., 2017).

Besides gender, there is a range of other social inequalities related to WASH. Caste relations have shown to facilitate or create barriers to sanitation interventions, related to cleaning, access to subsidies, latrine design, and purity issues (O'Reilly et al., 2017). People experiencing homelessness often face a denial of their rights to safe water and sanitation (Neves‐Silva & Martins, 2018). For people with disability, WASH services often do not meet specific needs for hygiene and privacy, or eliminate discrimination and abuse (Banks et al., 2019). A multicountry study reported that 23%–80% people with disabilities were unable to fetch water on their own, and those with more severe impairments had problems accessing the sanitation facilities used by other household members (Mactaggart et al., 2018). In many cases gender intersects with other social identifies such as age, sexual orientation, ethnic group, caste, disability, and this may exacerbate disadvantage (or expand advantage) (Crenshaw, 1989). For instance, displaced women and girls face particular challenges in access to safe and private facilities for menstrual hygiene management (Schmitt et al., 2017).

Awareness of these inequalities has resulted in implementation of WASH interventions that include mainstreaming of gender and social equality (GSE) considerations. While a large focus, in terms of both theoretical and empirical work, has been placed on gender inequalities, other forms of social exclusion related to WASH are also being increasingly addressed (WaterAid, 2010). WASH practitioners argue that such interventions will result in services that meet the needs of different groups, as well as challenge unequal power relations in society (Carrard et al., 2013). For example, adequate sanitation and hygiene facilities in schools are widely considered to facilitate girls' school participation and contribute positively to a sense of dignity and self‐esteem (Sommer et al., 2016). Easily accessible water sources are thought to increase economic opportunities and economic empowerment, as people spend less time and energy on unpaid work and have more time for productive or leisure activities. The time‐savings benefits of improved water access have long been recognised as reason alone to investment in improved water supply, even without demonstrable benefits on child survival health (Churchill et al., 1987). For example, Cairncross and Cliff (1987) demonstrated substantial opportunity costs of inadequate water supply for women, which affected time available for child‐care, food preparation, household hygiene, rest, and income generation. Moreover, household sanitation facilities or water on premises are thought to decrease risks of violence associated with open defecation or water collection (Geere et al., 2018; Jadhav et al., 2016). Consideration of gender and power relations within WASH interventions has also been shown to improve women's self‐confidence in intra‐household relations (Leahy et al., 2017), and participation in society, such as community‐level decision‐making (Sam & Todd, 2020).

Despite the wide range of GSE outcomes associated with WASH interventions, evidence has often been anecdotal, based on assumptions, or reported only in the grey literature. Funding agencies, governments, civil society organisations and academia alike have placed a greater emphasis on rigorous evaluation of technical and health outcomes of WASH interventions. This includes measuring provision or uptake of WASH‐related technology or behaviours such as safe water storage, hand‐washing with soap after using a toilet, toilet maintenance and similar (Parvez et al., 2018), or evaluating the relationships between access to inadequate WASH facilities and incidence of diarrhoeal diseases and other infectious diseases (Crocker & Bartram, 2016; Pickering et al., 2019).

Limited efforts to evaluate GSE outcomes may be related to the challenges of measuring social change, often a complex, nonlinear, context‐specific, and slow process (Hillenbrand et al., 2015). It can be difficult to trace clear causal pathways between intervention components and targeted outcomes. For instance, improvements in GSE outcomes may be cross‐sectoral, with difficulties attributing change directly to particular WASH components. Despite these challenges it is important to understand what kind of interventions are most often associated with better or worse GSE outcomes. A lack of attention to monitoring and evaluating changes in GSE outcomes or development of validated methodological approaches for evaluating GSE outcomes (UNESCO, 2019) has translated into gaps in understanding which intervention components contribute to the greatest positive impacts on GSE outcomes, as well as which interventions may lead or contribute to negative impacts that reinforce inequalities. These gaps in understanding are evident in the global policy discourse. For example, Sustainable Development Goal 6 “Clean Water and Sanitation” refers to the sanitation needs of women and girls, but has been described as “gender blind” due to the lack of gender‐sensitive targets (UN WOMEN, 2016). A comprehensive synthesis and greater availability of evidence of GSE outcomes resulting from WASH interventions is therefore needed to support WASH intervention design, implementation, and evaluation.

### 1.2 The intervention: Understanding gender and social equality in WASH interventions

In this review, we use *gender* to describe socially constructed identity and the related contextual and variable set of roles, behaviours, norms and responsibilities, while *sex* refers to a spectrum of biological differences. Although WASH interventions have often applied a female/male binary understanding of gender, there is a diverse spectrum of gender identities and gender expressions, including those who identify across or outside of the gender binary, and this group is described as gender minorities. Gender comprises part of a broader concept of social (in)equality and power hierarchies (Segnestam, 2018). Moreover, gender and other social identities such as age, sexual orientation, ethnic group, citizenship status, socio‐economic status, caste, disability, marital status are interdependent, and may intersect to exacerbate exclusion (Crenshaw, 1989). For instance, there is a particularly large burden on young girls and adolescents for water‐related responsibilities, while boys may be involved in water fetching for productive water uses (Thompson et al., 2011). It is important to note that local interpretations of what is meant by gender equality and other forms of social equality may be contested, adapted, and negotiated, which then influences engagement with the normative global discourse on gender equality. In a particular context, this may influence what components of a WASH intervention targeting GSE outcomes are culturally acceptable or relevant.

In the WASH sector, addressing gender and social equality has often focused on meeting practical needs (Moser, 1989), such as interventions that address people's needs based on gender and other socially constructed roles. This frequently involves instrumental approaches, whereby the focus is on “engaging women” to achieve other ends (e.g., such as engaging women to promote child health or economic development) (MacArthur et al., 2020). Alone these approaches are not viewed as adequate to address inequalities without addressing power issues, the burden of work, or similar (Cornwall, 2016; Hillenbrand et al., 2015). More recently, gender transformative development that addresses unequal power relations, structures and norms is being more widely taken up by actors in the WASH sector (MacArthur et al., 2020; Oxfam, 2020). These approaches focus on power dynamics between different social groups in varied social contexts and seek to address how these relations produce inequalities. For instance, interventions that address these considerations may result in more equal sharing of unpaid domestic and care responsibilities or increased opportunities for marginalised groups to use their voice in decision‐making. A key component of gender transformative approaches in the development sector is women's empowerment, which is understood as a complex process occurring at different levels, spaces and over time (Cornwall, 2016). However, gender transformative approaches aim to go beyond women's empowerment, emphasising working with both women and men to transform social relations towards more equitable arrangements.

To measure and evaluate change, interventions have sometimes been described in terms of their level of responsiveness to gender (and less commonly, social equality) aims. While a range of terms may be used to categorise outcomes (e.g., gender‐sensitive, gender‐responsive, gender integration), they are generally placed along a continuum (Pederson et al., 2014). At one end, interventions are gender‐blind and may exacerbate or exploit inequalities. In the middle, interventions may be inclusive of gender needs to varying extents, such as providing safe water supply or sanitation facilities, but may have a neutral impact on gender and social power relations. At the other end, interventions are aimed at transforming gender and social norms and relations. For instance, WaterAid developed a framework that categorises gender outcomes across the WASH system as ranging from harmful to inclusive, empowering and transformative (WaterAid, 2018). In this review, we use inclusive and transformative gender and social equality outcomes to capture two broadly defined categories of outcomes.

### 1.3 How the intervention might work

**Figure 1:**
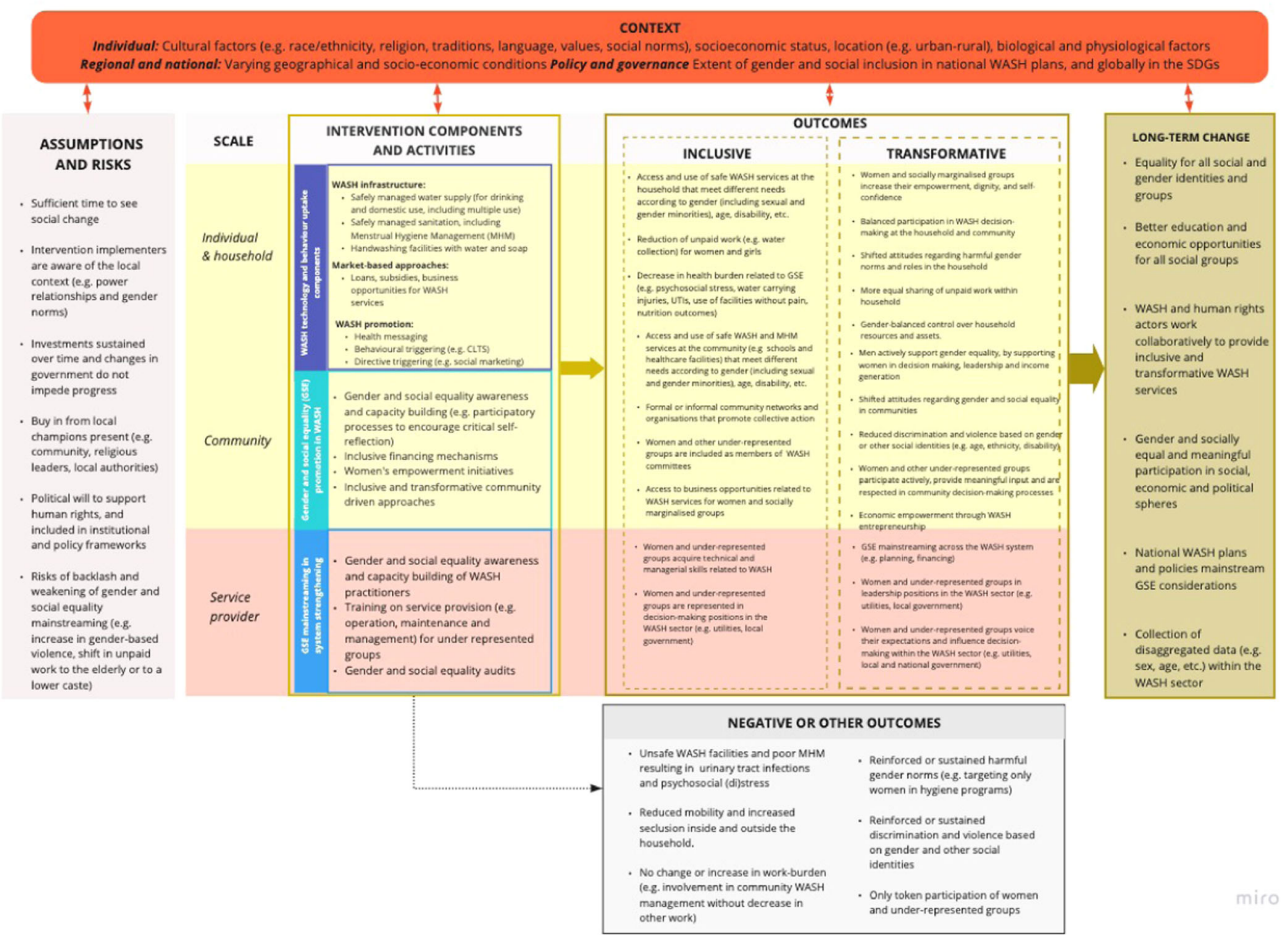
A draft theory of change (also available from: https://miro.com/app/board/o9J_ks7q_N8=/). *Source*: Authors

Our theory of change for promoting gender and social equality through WASH interventions is that implementation of various WASH technologies and promotion of behaviours, combined with GSE mainstreaming components, can lead or contribute to better access to services that meet the specific needs of all users (Figure 1). If GSE considerations go beyond meeting the needs of individuals to challenge power relations, WASH interventions will lead to or contribute to transformative changes that reduce inequalities related to WASH challenges and broader society. Together these outcomes will result in or contribute to long term changes in outcomes related to gender and social equality more widely, across different levels of society. These could be increases in the participation of women, girls and marginalised groups in public and economic life, better opportunities for education and livelihoods, and decreased discrimination and violence. At the same time, we acknowledge that these types of changes are complex, slow‐acting and nonlinear. Below, we describe WASH intervention components and resulting GSE outcomes illustrated in our theory of change in more detail.

#### 1.3.1 WASH interventions and their components

Water supply, sanitation or hygiene intervention components are sometimes grouped together (e.g., WASH) due to their inter‐dependent nature, particularly in rural settings. WASH interventions can be described with four main components: “how,” “what,” “where” and “for whom” (Waddington et al., forthcoming). “How” describes how the intervention is delivered, such as behaviour change approaches (e.g., triggering campaigns to end open defecation and similar). “What” describes the targeted WASH technology or practice (e.g., toilet usage, construction of water supply or hand‐washing stations). In the case of water supply, while a focus has been largely on safe drinking water, some interventions may go beyond meeting basic needs for drinking water and hygiene, to serve a range of uses including productive uses (e.g., livestock watering), known as Multiple Use of Water Services. In addition to supply driven approaches, WASH interventions can also involve the use of market‐based approaches to strengthen supply and demand, such as through training of local vendors, or smart subsidies and loans to households to promote uptake of WASH services (USAID, 2018).

“For whom” refers to the targeted participants. Most WASH interventions attempt to improve service provision for households, however interventions may target individuals, entire communities, service providers and authorities at national and subnational levels. Intervention components may be adapated to meet the needs of different groups, such as ensuring menstrual hygiene management in sanitation facilities. WASH interventions may also take place at the service‐provider or regulator level (e.g., local government overseeing service provision and setting up policy and accountability mechanisms) level as part of WASH system strengthening. WASH systems refers to all the social, technical, institutional, environmental and financial elements, actors, relationships and interactions that impact service delivery (Huston & Moriarty, 2018). “Where” describes the targeted location of the intervention such as the household, community (e.g., marketplaces, religious buildings), school or health facilities. Many aspects of WASH service delivery are cross‐sectoral, including housing, education, or health sectors, which can lead to complex arrangements with no clear governance structure. An example is WASH in‐school interventions, which target WASH services in schools to improve health and education outcomes together, which generally involve stakeholders from both WASH and education sectors (Deroo et al., 2015).

WASH interventions are increasingly using *GSE mainstreaming* components in their designs to ensure that they are inclusive of the needs of all users and contribute to GSE outcomes. Mainstreaming refers to addressing GSE considerations across development, planning, implementation, and evaluation of a WASH intervention (but it may be carried out to varying extents in different types of interventions). GSE mainstreaming is often viewed as having the dual purpose of improving the sustainability and effectiveness of the technical and health outcomes (e.g., such as uptake and sustained use of technologies or specific behaviours), as well as to promote positive change in GSE outcomes. Regardless of whether a WASH intervention includes intentional mainstreaming, it will still have social (and gendered) outcomes. Such an intervention may still lead to positive GSE outcomes, but no change in outcomes or regression with reinforced inequalities is also possible (Taukobong et al., 2016). This indicates the importance of intentional mainstreaming to influence these in the direction of inclusion and equality.

In this review we define WASH interventions as complex interventions because they are comprised of multiple components (show intervention complexity) and have multiple causal pathways and feedback loops (pathway complexity) (see Figure 1). In addition, they also often target multiple participants, groups, and organisational levels (population complexity), require multifaceted implementation strategies to boost adoption and uptake (implementation complexity) and are implemented in multidimensional settings (contextual complexity) (Guise et al., 2017).

#### 1.3.2 GSE outcomes

We define GSE outcomes as inclusive and transformative. Inclusive WASH outcomes are those that relate to the specific WASH needs and barriers of different social groups (Hillenbrand et al., 2015). For instance, these interventions may involve female‐friendly school toilets (e.g., modifications to ensure adequate menstrual hygiene management facilities) to meet girls' menstrual hygiene needs (Schmitt et al., 2018; UNICEF WaterAid & WSUP, 2018), toilets adapted to people with disabilities or toilets that are adapted to religious or cultural practices. Inclusive WASH outcomes may involve provision of water at more convenient locations, such as on premises (e.g., within the household property) to reduce women's time and physical burden spent collecting water, provision of water and sanitation at healthcare facilities to improve maternal outcomes, or provision of sanitation in public spaces such as schools and marketplaces. Beyond infrastructure design, such intervention components may include sharing of information, fair tariff structures, inclusive operating time, etc.

To capture different types of transformative outcomes described in our theory of change we applied Rowland's framework of power (1997) (Table 1). Transformative approaches address social causes of being unable to access and benefit from WASH, and seek to transform harmful power dynamics, norms and relations such as unequal distribution of unpaid work in the household. For example, while provision of a safe water source on premises can reduce the amount of time someone needs to collect water, it does not change their status in the household or community. Any time savings may lead to expectations to conduct other unpaid work. In contrast, men assuming roles traditionally assigned to women may indirectly support women's participation and empowerment in other domains, such as having time to contribute to water governance. Transformative outcomes also relate to women or marginalised group gaining greater control of their lives, for example, obtaining expertise in managing a water source, acquiring land tenure documentation for a water source, or gaining financial autonomy through WASH entrepreneurship.

**Table 1:** Types of power with examples of transformative GSE outcomes relevant for WASH sector

Power types	Description	Transformative GSE outcomes
Power within	A person's or group's sense of self‐worth, self‐awareness, self‐knowledge, and aspirations, which are also associated with agency and shaped by social norms and gendered relations	Increased self‐efficacy, self‐worth and self‐confidence, ability to claim one's right to safe water, sanitation, and hygiene
		Shifted perceptions towards gender and social equality, for example, men actively support women in decision‐making and leadership
Power to	Ability to make decisions, act and to realize one's aspirations. It is directly related to the agency dimension of empowerment and is frequently measured in terms of individual skills, capacities	Balanced participation in WASH decision‐making in the household and community More equal sharing of unpaid work within the household and community Acquiring technical and managerial skills related to WASH services
		Engagement of under‐represented groups in design processes and WASH trainings
Power over	Control over resources (e.g., financial, physical, personal networks and people)	Under‐represented groups obtain leadership positions in the WASH sector (e.g., utilities, local government) Economic empowerment through access to financial resources (e.g., cooperative memberships, loans, subsidies) and business opportunities for WASH services Greater access to formal and informal networks that support WASH‐related activities. Opportunities to voice expectations and influence decision‐making within the WASH sector (e.g., citizen accountability to local and national government)
Power with	Involves collaborative and collective power with others through mutual support	Community organisations that support women and marginalised groups in decision‐making, leadership and income generation related to WASH‐activities Informal social structures and networks that promote collective agency Development of cross‐sectoral (beyond WASH) initiatives and structures that focus on transformative GSE outcomes

*Note*: Adapted from Rowlands (1997) and Indarti et al. (2019).

In addition, there may be neutral, negative or other unexpected outcomes resulting from WASH interventions, as shown in Figure 1. In some cases, these may exacerbate inequalities related to WASH. For example, a sanitation intervention may lead to increased exposure to violence and discrimination if facilities are constructed without considering the needs of women and vulnerable groups, lead to backlash related to challenging social norms, or increase the burden of unpaid work (e.g., refilling handwashing stations). Other unintended harmful effects may include unpaid domestic labour shifted to the elderly or to a lower caste. Even when implementers consult with community leaders about socially acceptable ways of working with the community, WASH interventions may lead to increased resistance towards gender equality both at the household (e.g., (re)distribution of work) and community level (e.g., decision making in WASH governance).

#### 1.3.3 Context, assumptions and risks

The process of social change is complex, nonlinear, and it can take a long time to observe change. These processes are highly contextual and dependent on social, gender, cultural, economic, ecological and institutional factors at individual, household, community, and institutional spheres (Carrard et al., 2013). Thus, no intervention leads to positive GSE outcomes in all contexts and outcomes. The outcomes of WASH interventions are also dependent on a set of assumptions, such as continuing investments and political will to support the kind of WASH interventions that lead to GSE outcomes. For instance, in some settings discriminatory policies or laws may be put in place which hinder progress, despite a well‐designed intervention (especially at the level of service provision). There are also risks associated with addressing GSE due to possible backlash. For example, WASH interventions targeting increased decision‐making opportunities in one setting, or reduction of gender‐based violence, may lead to increase in another setting, or to women having less agency regarding their mobility both in and outside the household. There may also be unintended consequences of WASH technology provision as a result of interactions with social norms. For example, Rogers (2005) documented in Egypt that improved village water supplies were viewed suspiciously by villagers, who thought the taste of chlorine in the water was part of a government sterilisation programme. In addition, women preferred to collect surface water from canals where they could socialise with other women.

Finally, each of the outcomes includes an intermediate step to reaching that outcome (such as capacity building for improving employment opportunities or similar) but this could not be represented in Figure 1.

### 1.4 Why it is important to do this review

There is a growing interest in transformative WASH interventions because of their potential for delivering impact (Oxfam, 2020; WaterAid, 2018). A key message from the UN WOMEN Expert Group Meeting on Gender Equality and Water, Sanitation and Hygiene was as follows: “Taps and toilets are not enough. To realize transformational WASH outcomes, governments must enable women's voice, choice and agency” (UN WOMEN, 2017). In parallel, there is a growing emphasis on developing tools for collecting data on gender outcomes and disaggregating data by sex, age, ability status and other factors (Miletto et al., 2019).

Despite the interest in these outcomes, evaluation practice in the WASH sector has placed more focus on technical and health outcomes, such as technical standards for water sources, or evaluating diarrhoea prevalence, leaving gaps relating to evaluating gender and social equality outcomes (Loevinsohn et al., 2015; Mackinnon et al., 2019). This gap can translate into a lack of budget line items and prioritisation by stakeholders.

Most existing reviews on WASH have no explicit focus on gender, education or other social outcomes. Some reviews account for gender only as a contextual factor in the WASH intervention design (De Buck et al., 2017) or adoption (Hulland et al., 2015). The past and ongoing reviews that explicitly focus on social outcomes have a relatively narrow scope (only one WASH component such as menstrual hygiene management) or one specific group (e.g., girls in schools)) and some of them were conducted more than seven years ago (Birdthistle et al., 2011; DFID, 2013; Hennegan & Montgomery, 2016; Hennegan et al., 2019; Jasper et al., 2012; Munro et al., 2020; Sumpter & Torondel, 2013).

An evidence‐and‐gap map (EGM) (Waddington et al., 2018) compiled systematic reviews and impact assessments and mapped outcomes such as psycho‐social health, education, labour market outcomes, safety and income, consumption or poverty (see https://gapmaps.3ieimpact.org/evidence-maps/water-sanitation-and-hygiene-wash-evidence-gap-map-2018-update). The EGM did not include primary study evidence that used methods other than quantitative approaches, or undertake synthesis of findings from included impact evaluations. The impact studies included in the EGM, will be assessed for eligibility in the current review.

Thus, this review will provide a much‐needed synthesis of effectiveness of complex WASH interventions in contributing to GSE outcomes, facilitating better conceptualisation of GSE and WASH links as well as contributing to development of measurement tools to more accurately evaluate the GSE outcomes. The development of different measurement tools is already happening (e.g., Empowerment in WASH Index [EWI]: https://www.sei.org/projects-and-tools/projects/ewi-empowerment-in-wash-index/); or WASH Gender Equality Measure (WASH‐GEM): https://waterforwomen.uts.edu.au/wash-gem-piloting-in-cambodia-and-nepal/) and the review can directly inform this ongoing work.

## 2 OBJECTIVES

This review aims to comprehensively and transparently synthesise evidence on gender and social equality outcomes in complex WASH interventions. We also aim to develop and test a set of hypotheses about causal relationships between WASH intervention components and outcomes and related to our theory of change. Our aim is to advance evaluation practices in the WASH sector by providing methodological advice on how to include, assess and measure GSE outcomes. Additionally, we will map definitions of different outcome measures and provide guidelines on this. The findings will be of use for decision makers in policy and practice allowing them to more effectively design and implement gender and social equality mainstreaming in WASH interventions and strategies and learn from best practices. By describing methodological deficiencies in relevant primary research (see section *Assessment of risk of bias in included studies*), we will provide guidance and best practice examples for future primary research on the subject.

The review questions are:

**Review question 1:** *What are the impacts of complex WASH interventions on gender and social equality outcomes in low‐ and middle‐income countries?*

**Review question 2:** *What are barriers to or enablers of change in these outcomes?*

**Review question 3:** *Under which conditions do WASH intervention (components) lead to a change in GSE outcomes?*

**Review question 4:** *How are GSE outcomes measured in the literature?*

## 3 METHODS

This review follows Campbell Collaboration policies and guidelines (The Campbell Collaboration, 2019).

### 3.1 Stakeholder engagement

Principles of stakeholder engagement and co‐design will be applied used throughout the review process to improve the rigour of research, maximise acceptance and legitimacy, provide a strong science‐policy link (Land et al., 2017) and facilitate communication of findings (Haddaway & Crowe, 2018).

We comprehensively mapped stakeholders that work in the WASH implementation and policy space, and closely linked stakeholders working on gender and social equality more broadly. A suite of complementary processes was used to identify and map stakeholders (e.g., snowballing and systematic searching). The resulting stakeholder map will also be used for the communication of review findings. Identified key stakeholders, such as representatives of funding agencies and civil society organisations engaged in WASH interventions, and researchers with expertise on a range of WASH outcomes, were engaged in the codesign of the systematic review protocol, review scope and questions, definitions and a theory of change to model the link between intervention components, the context and GSE outcomes (see Figure 1). The engagement occurred via two online workshops in May and June 2020. In addition and to obtain input of wider community, we invited stakeholders to comment on a previous version of the review protocol that was publicly available on the website of Stockholm Environment Institute and shared via Sustainable Sanitation Alliance network (https://www.susana.org), the Rural Water Supply Network and other online communities of WASH practitioners between 16 July and 3 August 2020. Stakeholders' inputs on the protocol and our responses are available here: https://www.sei.org/projects-and-tools/projects/advancing-evaluation-of-gender-equality-outcomes-in-wash/.

### 3.2 Criteria for considering studies for this review

Below we describe the eligibility criteria. For all the review questions we will apply the same eligibility criteria (except for Types of studies, see below for details).

#### 3.2.1 Types of studies

In order to answer review question 1, we will consider quantitative research with experimental designs (with random assignment), quasi‐experimental designs and natural experiments, which are able to address confounding:

- Randomised controlled trials, with assignment to intervention or “encouragement” to intervention at individual or cluster level.
- Quasi‐experimental designs with nonrandom assignment, using methods such as naïve and statistical matching on baseline data, and double‐difference analysis of data pre‐ and posttest data.
- Natural experiments using methods such as regression discontinuity design to construct comparison groups, where assignment is determined at pretest by a cut‐off on an ordinal or continuous variable (White & Sabarwal, 2014)).
- Pipe‐line designs, where individuals or groups are followed over time and compared to comparisons who are eligible for intervention at a later date.
- In addition, pre‐post studies will be included in the particular case of immediate outcomes such as time‐use or time‐savings, for which the expected effect is large and confounding is unlikely (Victora et al., 2004).
- Mixed‐method study designs will be considered that examine results along the causal pathway, reporting intermediate and endpoint outcomes.

In order to answer research question 2, all qualitative and mixed‐method study designs will be considered, regardless of whether the study design includes an explicit comparator (whether from a separate group or a pretest). All eligible studies included under research questions 1 and 2 will be eligible to answer research question 3 and 4. No commentary papers, theoretical or modelling studies will be included. We will include studies regardless of their publication status and their electronic availability.

#### 3.2.2 Types of participants

All types of study participants (from different gender and social identities, age groups and across rural and urban settings) will be included but restricted to those in low‐ and middle‐income countries (LMICs). We will use the LMIC definition provided by the World Bank including low‐, lower‐middle and upper‐middle income economies from their classification for year 2021 (see https://datahelpdesk.worldbank.org/knowledgebase/articles/906519-world-bank-country-and-lending-groups).

#### 3.2.3 Types of interventions

All types of interventions providing water, sanitation and hygiene software and hardware technologies implemented in both rural and urban settings are eligible for the review. Following Waddington et al. (2018), these include direct hardware provision, behavioural change communication (such as health messaging, psychosocial “triggering”), market‐based approaches (such as subsidies for WASH consumers and microloans or training for producers), GSE promotion in WASH, GSE mainstreaming in system strengthening, systems‐based approaches (such as programmes to empower women in WASH decision making and governance, privatisation or nationalisation of water supply and sewage systems, or decentralised provision, e.g., community‐driven development) and a combination of two or more components are relevant for this review.

WASH interventions may also take place at the service‐provider or regulator (e.g., local government overseeing service provision and setting up policy and accountability mechanisms) level as part of WASH system strengthening. Interventions focusing on irrigation or water resources management are beyond the scope of the review.

#### 3.2.4 Types of outcome measures

##### Primary outcomes

Any types of GSE outcomes resulting from WASH intervention(s) will be included and categorised into inclusive and transformative outcomes as described above in the Theory of Change. This includes, for example, level of or change in empowerment, such as self‐efficacy, voice, participation, agency and decision‐making related to WASH or more generally (e.g., participation in community‐based decision‐making on WASH or more generally), gender‐based violence, discrimination, injury (e.g., pedestrian traffic injury), attack by wild animals, mental health and other psychosocial outcomes (e.g., self‐esteem), time‐use, work burden, access to jobs, access to leisure and sleep, ownership and control of assets, and changes in behaviour such as increased use of WASH facilities among different groups. Additionally, we will record any adverse and unintended effects of WASH interventions that exacerbate inequalities or negatively affect GSE. If reported, evidence of lack of change will also be recorded. We will exclude outcomes relating to infectious disease and poor water quality, such as diarrhoea and stunting, which are covered extensively in other reviews, but will include health outcomes related to GSE and arising from gender roles and social norms such as musculoskeletal injuries and reduced nutritional status from water carrying, infections from poor menstrual hygiene management and psychosocial stress from poor sanitation facilities. We will include any type of measures of eligible outcomes.

##### Secondary outcomes

We will record any type of intermediate outcomes, thatis, outcomes that are precursors of (or a necessary condition for) empowerment or other gender and social equality outcomes such as change in level of knowledge, capacity and/or awareness. Studies that include only secondary outcomes will be considered eligible and evidence from these studies will inform theory of change development (see Section 3.4.11).

##### Duration of follow‐up

All durations of follow‐up are eligible for inclusion, including multiple durations of follow‐up in any single study.

##### Time frame

Due to the wide‐ranging and comprehensive scope of the review, we will include publications from January 2010 to September 2020 to ensure feasibility. Publications prior to 2010 will be excluded.

##### Types of settings

We will include WASH interventions implemented in both rural and urban settings including households, schools, health facilities, community spaces or workplace settings, and restricted to LMICs.

##### Eligible languages

We will include studies in English, Spanish and French (as per skillset of the review team).

### 3.3 Search methods for identification of studies

We will use a multipronged search strategy. All the searches, as justified above, will be done for literature published after 2010.

#### 3.3.1 Electronic searches

##### Bibliographic databases

We will search for literature in English in several bibliographic databases and platforms (using subscriptions of Stockholm University and University of Sussex) including:

1. Web of Science Core Collections
2. PubMed
3. Cumulative Index of Nursing and Allied Health Literature (CINAHL)
4. WHO Global Health Library
5. Econlit
6. Electronic Theses Online Service (ETHOS)
7. Digital Access to Research Theses (DART)
8. ProQuest: Dissertations and Theses
9. Networked Digital Library of Theses and Dissertations (NDLTD)
10. The Trials Register of Promoting Health Interventions (TRoPHI)
11. APA PsycINFO
12. APA PsycArticles
13. Sociological abstracts
14. OpenGrey
15. Education Resource Information Center (ERIC)
16. International Initiative for Impact Evaluation (3ie)

In the final report we will detail for each search source which interface was used, and which search settings were applied. Examples of search strategies for selected sources can be found in the Supporting Information.

Table 2 shows two search substrings with terms related to WASH interventions and GSE outcomes (shown as formatted for Web of Science and to be adapted for other search sources depending on their search facilities). The full search string combines the two substrings with Boolean operator “AND.” Search terms were compiled with stakeholders' input.

**Table 2:** Search string

Substring 1: WASH‐related terms		Substring 2: GSE‐related terms
toilet* OR latrine* OR watsan OR sanita* OR sewage OR sewerage OR wastewater* OR "waste water" OR (water NEAR/2 suppl*) OR (water NEAR/2 access) OR "water management" OR (water NEAR/2 drinking) OR (water NEAR/2 scarcity) OR handwash* OR "hand wash*" OR soap$ OR "WASH intervention*" OR "piped water" OR "tippy tap*" OR (water NEAR/2 point) OR (water NEAR/2 service) OR (water NEAR/2 security) OR (water NEAR/2 insecurity) OR "open defecat*" OR (hygiene NEAR/2 promo*) OR "water filter" OR "water pump*" OR "menstrual poverty" OR "period poverty" OR handpump* OR "hand pump*" OR (water NEAR/2 collection) OR "water committee*" OR "water well*"	AND	gender* OR discrimination* OR *equalit* OR *equit* OR inclusive OR "sexual minorit*" OR transgender OR femin* OR masculin* OR menstr* OR menses OR UTI OR "urinary tract infection" OR uro$genital OR pain OR *empower* OR school* OR educat* OR violen* OR psychosocial OR "psycho‐social" OR "psycho social" OR "psychological *stress" OR "mental health" OR dignity OR fear* OR taboo* OR elder* OR disabilit* OR caste OR "social class*" OR daughter* OR girl* OR boy$ OR child* OR prestig* OR sham* OR stigma OR privacy OR voice* OR well$being* OR povert* OR "unpaid labor" OR "unpaid labour" OR livelihood* OR income OR fetch* OR esteem* OR "social capital" OR "land tenure" OR leadership OR time$saving OR "transactional sex" OR musco$skeletal OR musculoskeletal OR wife OR wives OR husband$ OR "decision‐making" OR "decision making"

*Note*: This search yielded 27500 results in Web of Science Core Collections for Topic search (including search on title, abstract and keywords) with a subscription of the University of Stockholm. The search was performed on 16 October 2020 for literature published between 2010 and 2020, and filtered to English, Spanish and French languages only.

#### 3.3.2 Searching other resources

##### Specialist websites

Searches will be performed across a suite of relevant organisational websites (see Table 3). The list of the relevant websites is compiled with inputs from stakeholders. These searches will be particularly important for capturing grey literature. Websites that contain information available in bibliographic databases will not be searched. Each website will be hand‐searched for relevant publications.

**Table 3:** Specialist websites with details of search languages

	Organisation	Website	Search language
1	African Development Bank's Africa Water Facility	https://www.afdb.org/en/topics-and-sectors/initiatives-partnerships/african-water-facility	English, French
2	The United Nations Children's Fund (UNICEF)	https://www.unicef.org/	English, Spanish, French
3	The United Nations Development Programme (UNDP)	https://www.undp.org/	English, Spanish, French
4	UN Women	https://www.unwomen.org/en/digital-library/publications	English, Spanish, French
5	The United Nations Population Fund (UNFPA)	https://www.unfpa.org/	English, Spanish, French
6	The United Nations Human Rights (OHCHR)	https://www.ohchr.org/EN/Issues/WaterAndSanitation/SRWater/Pages/AnnualReports.aspx	English, Spanish, French
7	The Deutsche Gesellschaft für Internationale Zusammenarbeit GmbH (GIZ)	https://www.giz.de/en/html/index.html	English
8	The United States Agency for International Development (USAID)	https://www.usaid.gov/developer/development-experience-clearinghouse-dec-api	English
9	WaterAid	https://washmatters.wateraid.org/	English
10	Oxfam International	https://policy-practice.oxfam.org.uk/	English, Spanish, French
11	Oversees Development Institute (ODI)	https://www.odi.org/	English
12	The World Bank (WB)	https://www.worldbank.org/	English
13	The Department for International Development (DFID)	https://www.gov.uk/government/organisations/department-for-international-development	English
14	The Swedish International Development Cooperation Agency (Sida)	https://www.sida.se/English/	English
15	CAF—Development Bank of Latin America	https://www.caf.com/en/	Spanish
16	Inter‐American Development Bank (IADB)	https://www.iadb.org/es/sectores/iniciativas-agua	English, Spanish
17	Care	https://insights.careinternational.org.uk/	English
18	Femme International	www.femmeinternational.org	English
19	SNV	https://snv.org/sector/water-sanitation-hygiene	English
20	Menstrual Hygiene Day	https://menstrualhygieneday.org/resources-on-mhm/resources-mhm/	English
21	PATH	https://www.path.org/	English
22	International Disability Alliance	http://www.internationaldisabilityalliance.org/	English
23	Programme Solidarité Eau	https://www.pseau.org/fr	French
24	Sustainable Sanitation Aliance (SuSanA)	https://www.susana.org/	English
25	SuSanA Latin American chapter	https://www.susana.org/en/knowledge-hub/regional-chapters/latinoamerica-chapter	Spanish
26	Action contre la Faim	https://www.actioncontrelafaim.org	French
27	Sanitation Learning Hub	https://sanitationlearninghub.org/	English
28	Water for Women	https://www.waterforwomenfund.org/en/index.aspx	English
29	WaterAid: Inclusive WASH	https://www.inclusivewash.org.au/	English
30	iDE	www.ideglobal.org	English
31	SIMAVI	https://simavi.org/	English
32	Plan International	https://plan-international.org/	English
33	Water and Sanitation for the Urban Poor (WSUP)	https://www.wsup.com/	English
34	International Water Association (IWA)	https://iwa-network.org/	English
35	Multiple Use of Water Services	https://www.musgroup.net/	English
36	PSI	https://www.psi.org/practice-area/wash/	English
37	IRC	https://www.ircwash.org/	English
38	ONGAWA Ingeniería para el Desarrollo Humano	https://ongawa.org/	Spanish
39	The Water Supply and Sanitation Collaborative Council (WSSCC)	https://www.wsscc.org/	English
40	Sanitation and Water for All (SWA)	https://sanitationandwaterforall.org/	English
41	Enterprise in WASH	http://enterpriseinwash.info/research-outputs/	English
42	BRAC	http://www.brac.net/wash	English
43	World vision	https://www.worldvision.org/	English
44	Organisation for Economic Co‐operation and Development (OECD)	https://www.oecd.org/water/	English
45	FESAN, Federacion Nacional de Cooperativas de Servicios Sanitarios Rurales Chile Ltda	http://fesan.coop/	English, Spanish
46	Engineering for change	www.engineeringforchange.org	English
47	European Civil Protection and Humanitarian Aid Operations (ECHO)	https://ec.europa.eu/echo/who/accountability/annual-reports_en	English
48	Australian Department of Foreign Affairs and Trade (DFAT)	https://www.dfat.gov.au/	English
49	Office of US Foreign Disaster Assistance (OFDA)	https://www.usaid.gov/who-we-are/organization/bureaus/bureau-democracy-conflict-and-humanitarian-assistance/office-us	English
50	Global Waters	www.globalwaters.org	English
51	EcoSanRes	http://www.ecosanres.org/publications.htm	English
52	Sanitation Updates blog	https://sanitationupdates.blog/	English
53	Water Currents	https://www.globalwaters.org/resources/water-currents	English
54	United Nations Evaluation Group (UNEG)	http://www.uneval.org/evaluation/reports	English

Additionally, bibliographies of relevant reviews identified during searching will be checked for relevant literature. We will ask stakeholders (including researchers) to provide relevant literature, including data from unpublished or ongoing relevant research.

##### Search engines

Searches will be performed in English, Spanish and French in Google Scholar, with simplified sets of search strings, combining both WASH and GSE terms. The first 1000 search results (Haddaway et al., 2015) will be extracted as citations using Publish or Perish software (Harzing, 2007) and introduced into the duplication removal and screening workflow alongside records from bibliographic databases.

##### Additional sources

Additional searches for eligible literature will be done in reference lists of eligible studies (included at full text) and bibliographies of relevant reviews (including evidence gap maps). We will also draw on the studies published in the WASH evidence and gap map (Waddington et al., forthcoming; Waddington et al., 2018). Furthermore, we will contact relevant experts and organisations for relevant research, unpublished or ongoing studies.

##### Testing comprehensiveness of the search

A list of 32 articles of known relevance to the review (a benchmark list) was screened against search results to examine whether the search strategy was able to locate relevant records (a benchmark list can be found in the Supporting Information). In cases where these articles were not found during the scoping exercise, search terms were examined to identify the reasons why relevant records were missed, and search terms were modified accordingly and until all the records from the benchmark list were picked up by the string. The final version of the search string picked all the articles from the list.

##### Assembling library of search results

Results of the searches in bibliographic databases and Google Scholar will be combined, and duplicates removed prior to screening. A library of search results will be assembled in a review management software EPPI reviewer (Thomas et al., 2020).

### 3.4 Data collection and analysis

#### 3.4.1 Description of methods used in primary research

We anticipate that our evidence base will include quantitative, qualitative and mixed method research, including impact assessments and other types of project evaluations. A number of studies have collected data on time‐use (usually due to improved water supplies) and we will use it to exemplify a variety of potential methods used in primary research to study this outcome. Devoto et al. (2012) collected time‐use survey data in the context of a randomised encouragement trial in Morocco. Klasen et al. (2011) analysed time‐use available in household survey data in Yemen using fixed effects estimation. Dickinson et al. (2015) measured time use due to improved sanitation in a cluster‐randomised control trial. Arku (2010) conducted a retrospective mixed‐methods before‐after study in Ghana of improved water supply, which collected reported average time‐savings from improved water, relative to recalled baseline information. The study also collected qualitative evidence on barriers to accessing water through focus group discussion and key informant interviews.

#### 3.4.2 Criteria for determination of independent findings

Multiple intervention groups will be carefully assessed to avoid double counting and/or omissions of relevant groups. Dependency of findings will be assessed at the data, publication and within publication levels. Sources of dependency at data level include publications by different authors using the same data. We will endeavour to group any studies based on the same dataset under a single study. Similarly, we will group multiple publications of the same analysis (e.g., working paper versus journal article) under a single study. Dependency at the within‐publication level, for example reporting of multiple effect estimates by follow‐up period, or reporting of multiple outcomes, will be addressed by not including multiple findings in any single analysis, so as to not weight the dependent findings more heavily in comparison with studies reporting only single findings. Thus, for example, where multiple follow‐ups are reported, cross‐study meta‐analysis will include a “synthetic effect” calculated as the sample‐weighted average across follow‐ups (Waddington & Snilstveit, 2009). However, within‐study analysis may still draw on the multiple follow‐ups using time‐series analysis. Alternatively, and in cases where there are sufficient data, we will use multilevel meta‐analysis. Where multiple outcomes are reported, for example overall time‐savings, and time‐use for reproductive health, production and leisure, the outcomes will be analysed separately.

#### 3.4.3 Selection of studies

Screening will be conducted at two levels: at the title and abstract level together, and at full‐text level in EPPI reviewer. Retrieved full texts of potentially relevant records will then be screened at full text, with each record being assessed by one experienced reviewer. As we expect, based on the scoping exercise, the search to yield a large number of records (>40,000), double screening will not be possible due to lack of resources. Nevertheless, we will make the process of screening more efficient through the innovative use of machine learning and other automation technologies in EPPI‐Reviewer. Specifically, a combination of machine learning assisted screening function (“priority screening,” that uses a machine learning algorithm to “learn” the scope of the review as records are manually screened) and modelling (bespoke machine learning classifiers) will be used to support and facilitate manual title and abstract screening and help devise empirically informed cut off point below which no manual screening will be done. A training set would be prepared from the records that were screened by at least two reviewers Machine learning functionality in EPPI Reviewer is a technology in development, but it showed good in performance in screening prioritisation (Tsou et al., 2020).

To assure consistency among all reviewers, consistency checking will be performed on a subset of records at the beginning of each screening stage. A subset of 600 title and abstract records and 120 full texts will be independently screened by all reviewers. The results of the consistency checking will then be compared between reviewers and all disagreements will be discussed in detail. Where the level of agreement is low (below c. 80% agreement), further consistency checking will be performed on an additional set of records and then discussed. Following consistency checking (i.e., when the agreement is above 80%), records will be screened by one experienced reviewer.

#### 3.4.4 Data extraction and management

We will extract data and meta‐data following theory of change components, including bibliographic information; study aims and design including location, data collection method, sample size, analytic approach; critical appraisal, details about intervention and implementation context; population details (including age, identity group(s), intersectionality and other types of moderators); outcomes and study findings (the outcome means and measures of variation in case of quantitative research, or first and second order constructs in case of qualitative research). This list will be expanded during the review process (and as part of framework synthesis, see Section 3.4.11). A draft coding scheme can be found in the Supporting Information.

We will make maximal use of data and where quantitative data are not reported in the form suitable for meta‐analysis (e.g., as means and standard deviations), we will perform necessary data conversions and calculate desired metrics (e.g., *SE*s can be computed from confidence intervals, *t* statistics, and *p* values).

Prior to starting with coding and data extraction, and to assure repeatability of data extraction and coding process, consistency checking exercise will be performed on a subset of records (up to 10%) independently extracted by all reviewers. All disagreements will be discussed among reviewers, and coding scheme will be clarified if needed. The data extraction will be then performed by a single reviewer. In a scenario where only a small number of studies is included for data extraction (≤20), dual data extraction (i.e., by two reviewers independently) will be performed. Discrepancies in data extraction between the reviewers will be resolved through a discussion.

#### 3.4.5 Assessment of risk of bias in included studies

Eligible studies will be subject to critical appraisal, where existing and validated tools will be used. For assessing risk of bias in quantitative randomised and nonrandomised studies we will use a tool (Waddington, forthcoming) that has been developed for WASH sector impacts evaluations, drawing on and extending Cochrane's approaches for individual randomised studies (Higgins et al., 2016), cluster‐randomised studies (Eldridge et al., 2016) and nonrandomised studies (Sterne et al., 2016). Overall, the assessment of quantitative studies will include an assessment of external and internal validity including confounding, selection bias, missing data, deviations from intended intervention, measurement error, bias in reporting, and sampling bias.

For qualitative studies we will follow Noyes et al. (2019) guidance focusing on the quality in the research, assessing methodological strengths and limitations (such as clarity of aims and research questions, congruence between research aims and design, the rigour of case/participant identification, sampling and data collection to address the question, appropriates of method application, the richness of findings, exploration of deviant cases and reflexivity of researches) (and more here: https://training.cochrane.org/handbook/current/chapter-21#section-21-8).

As a result of the critical appraisal process, we might categorise relevant studies as, for example, having a high and low validity. This information will be used in a sensitivity analysis during the synthesis stage of the review. Studies with unacceptably low validity may be excluded from the review. The cut off points for each of the categories will be decided during the appraisal process and based on the overall state of the evidence (this will be described in detail in the review report). Studies will not be excluded on the basis of reporting of the outcome data to avoid selective outcome reporting bias.

Prior to starting with this stage, to test the appraisal tools and assure repeatability of the appraisal process, consistency checking will be performed on a subset of records (up to 10%) with different study designs independently assessed by all reviewers. All disagreements will be discussed among the team, and assessment criteria will be clarified if needed. All the studies will be appraised by at least two reviewers.

#### 3.4.6 Measures of treatment effect

Given that we expect differences (in scale and type of data) in outcome reporting, to compare results of continuous measures we may use the standardised mean difference (SMD), and the odds ratio (OR) for binary measures.

#### 3.4.7 Unit of analysis issues

Each article and each study will have uniqe IDs. In case of multi‐arm studies, only intervention and control groups that meet eligibility criteria will be included and related relevant outcome data will be extracted.

#### 3.4.8 Dealing with missing data

Authors of the original studies will be contacted for missing information (if correspondence details are valid and available). Where information is not available, such as on pooled standard deviations, appropriate methods will be used to derive effect sizes from reported information such as *t* statistics (Borenstein et al., 2009).

#### 3.4.9 Assessment of heterogeneity

For quantitative data (and in case of sufficient number of studies with sufficiently large sample sizes), forest plots will be inspected visually to see the overlap in the confidence intervals for outcome data. *I* ^2^ statistic will be calculated to quantify relative heterogeneity across studies (recognising that this statistic produces uncertain assessment of heterogeneity in cases where a number of studies is low (von Hippel, 2015). *τ* ^2^ will be calculated as a measure of absolute heterogeneity.

#### 3.4.10 Assessment of reporting biases

To assess risk of reporting bias, the (contour enhanced) funnel plots will be inspected visually, and Egger's test or an appropriate alternative for binary data) will be performed on quantitative data.

To minimise the risk of reporting bias we are conducting extensive searches for both academic and grey literature.

#### 3.4.11 Data synthesis

We will conduct a mixed methods evidence synthesis with a sequential design (Heyvaert et al., 2017) where a theory built in the first stage, will be “tested” in the second stage. For each of the synthesis stages we will use different approaches as detailed below.

First, we will use framework synthesis (of mixed‐method and qualitative research studies) to (1) improve initial theory of change (Figure 1) with new understandings about the links between intervention, their components and outcomes (Kneale et al., 2018), and (2) identify barriers and enablers. Analysis of effect sizes (or meta‐analysis)‐will be used to answer Review Question 1. Meta‐analysis will be done to present forest plots of effect sizes with 95 percent confidence intervals by outcome, using Stata software. Finally, we will use Qualitative Comparative Analysis (QCA) and/or meta‐regression to explore heterogeneity and test hypothesis from the theory of change to answer Review Question 3 (see next section).

Framework synthesis (Brunton et al., 2015, 2020; Macura et al., 2019) is a method for organising and synthesising diverse types of evidence as it can accommodate qualitative and mixed method studies. It can be used for studying complex interventions, while supplying (different types of) evidence across longer causal chains (Kneale et al., 2018). Framework synthesis is composed of six analytical stages including (1) familiarisation with the data; (2) framework creation or selection; (3) indexing of data according to a framework; (4) charting or rearranging the data according to the framework and potentially framework modification; (5) mapping and (6) interpretation. The *familiarisation* and *framework selection* stages were completed during the protocol drafting process as reviewers got familiar with the topic under study and drafted (along with the stakeholder input) theory of change (see Figure 1). In the next step and at the *indexing* stage, the review team will perform searches, screening, data extraction (informed by the draft theory of change and as described in the previous sections of this protocol) and identify main characteristics of relevant studies. In the *charting* stage, characterised studies will be further grouped into categories and themes will be derived from the data. At the *mapping* stage derived themes will be considered in the light of the original research question and we will investigate how derived themes relate to each other and to the theory of change that can be expanded with new themes at this stage. Based on the number of studies included at this stage, we will be able to decide the next synthesis step (see below). The studies will not be categorised and selected for the next stage on the basis of their results, however. At the *interpretation* stage derived themes will be considered in the light of the wider research literature.

In a scenario where two or more studies report similar types of quantitative outcomes, we will perform a meta‐analysis, where pooled effect sizes will be estimated using random effects and weighted appropriately to summarise the impact of the intervention. Where there are not more than a single study providing evidence for a particular outcome, we will present effect sizes in forest plots. In one meta‐analysis we will combine and compare GSE outcomes of the same type of WASH intervention (components) (e.g., outcomes of WASH infrastructure provision will not be compared with health messaging interventions).

We will test links from the (now expanded) theory of change and explore heterogeneity among studies using Qualitative Comparative Analysis (QCA) and/or meta‐regression. This step can increase the robustness of the claims made in the theory of change. QCA is a method for identifying (necessary and sufficient) relationships in data. In a review setting, QCA can identify or test links between participants, intervention (components) and context that may be associated with or trigger a successful outcome (Kahwati et al., 2016). QCA is valuable as it can examine complexity within small datasets where number of studies or examples of a phenomenon is small, but number of variables that might explain a given outcome or differences in study findings is large (Kneale et al., 2018; Thomas et al., 2014). The unit of analysis is not an individual study but a set or a configuration of intervention (components), participants and contextual characteristics that together lead (or not) to the outcome of interest. These configurations or combination of different factors are called conditions in QCA (James Thomas et al., 2014). QCA includes six stages: (1) building the data table describing set of conditions and outcomes for each study; (2) constructing a truth table and assigning studies with same configurations of conditions to sets; (3) resolving contradictory configurations where sets of studies with identical configurations of conditions lead to different outcomes; (4) Boolean minimisation to analyse the truth table, finding solutions which encompass as many studies as possible; (5) consideration of the “logical remainders cases” or configurations without any cases; and (6) interpretation of the solution in the light of studies included in the solution, the review questions and theory of change that guided the review (Thomas et al., 2014). The conditions will be identified in the primary studies and during indexing and mapping stage of the framework synthesis. Conditions could be factors that may act as a barrier or a facilitator (sometimes both at the same time and depending on context). Any identified condition thus can be reformulated into a hypothesis that can be tested via QCA. For example, a condition for equality could be that men's support increases women's engagement in WASH‐related decision making. A hypothesis to be tested is in this case is: Does men's support (=condition) increase the likelihood that women are invited into decision boards (=outcome)? Through QCA we could then obtain following answers: *yes*, *no*, or *depending on a specific context*.

Similarly, and in a scenario where enough studies report similar types of quantitative outcomes, meta‐regression could be performed as well to explore the heterogeneity and test hypotheses from the theory of change. The moderators for this analysis will be sourced from the theory of change (amended at the previous synthesis step). We will describe in which cases we will choose QCA over meta‐regression in detail in the review report.

#### 3.4.12 Sensitivity analysis

Sensitivity analysis will be performed during the synthesis stage to understand if results of the synthesis depend on the methodological rigour and susceptibility to bias of included studies. If there are sufficient data, we will run separate meta‐analyses for randomised controlled trials and quasi‐experimental studies. These two types of studies can be later combined in one meta‐analysis and results of these two analyses will be compared.

The final report will include a refined theory of change and a description of how intervention components contribute to or result in a change in specific GSE outcomes, an overview of different barriers and enablers to a change in outcomes and ways to measure these outcomes, an assessment of possible knowledge gaps and clusters (produced by cross‐tabulating extracted data from key variables (e.g., type of outcome by type of intervention(component)) and corroborating these findings with stakeholders) that may constitute priority topics for primary research, and a discussion of tentative policy implications of the review findings.

#### 3.4.13 Subgroup analysis and investigation of heterogeneity

Heterogeneity will be analysed using subgroup analysis, whereby gender and social equality outcomes for different population groups (such as women and men or rural, per‐urban and urban populations and similar) are reported. The study will also investigate heterogeneity in integrated synthesis using QCA.

#### 3.4.14 Treatment of qualitative research

As noted above, qualitative research will be incorporated to answer review questions 2 and 3.

## CONTRIBUTIONS OF AUTHORS

- *Content*: Sarah Dickin, Naomi Carrard, Louisa Gosling, Lewnida Sara, Marni Sommer, Hugh Sharma Waddington.
- *Systematic review methods*: Biljana Macura, James Thomas, Karin Hannes, Hugh Sharma Waddington.
- *Statistical analysis*: Biljana Macura, James Thomas, Hugh Sharma Waddington.
- *Information retrieval*: Laura Del Duca, Adriana Soto.

## DECLARATIONS OF INTEREST

Authors declare no conflict of interest. Reviewers will be prevented from taking part in inclusion decisions or validity assessment of articles they authored.

### PRELIMINARY TIMEFRAME

Approximate date for submission of the systematic review: December 2021.

### PLANS FOR UPDATING THIS REVIEW

At the moment we do not plan any updates due to limited resources.

## SOURCES OF SUPPORT

### Internal sources

This review project is co‐funded by Stockholm Environment Institute.

### External sources

- This review project is funded by the Centre of Excellence for Development Impact and Learning (https://cedilprogramme.org/funded-projects/programme-of-work-1/gender-and-social-outcomes-of-wash-interventions/).

## Supporting information

Supporting information

## Appendix

**Table:**

Title	Methodological expectations of Campbell Collaboration intervention reviews: Conduct standards
Authors	The Methods Group of the Campbell Collaboration
DOI	10.4073/cpg.2016.3
No. of pages	23
Last updated	28 October 2019
Citation	The Methods Group of the Campbell Collaboration. Methodological expectations of Campbell Collaboration intervention reviews: Conduct standards Campbell Policies and Guidelines Series No. 3 DOI: 10.4073/cpg.2016.3
ISSN	2535‐2458
Copyright	© The Campbell Collaboration This is an open‐access article distributed under the terms of the Creative Commons Attribution License, which permits unrestricted use, distribution, and reproduction in any medium, provided the original author and source are credited.
Acknowledgement	Adaptations on MECIR Version 2.2 Conduct Standards (Chandler, Churchill, Higgins, Lasserson, & Tovey, 2012) October 2019‐ updated to remove section numbers for Cochrane Handbook since these are out of date with the new Cochrane Handbook published in October 2019
Editor in Chief	Vivian Welch, University of Ottawa, Canada
Chief Executive Officer	Howard White, The Campbell Collaboration
Managing Editor	Chui Hsia Yong, The Campbell Collaboration
	The Campbell Collaboration was founded on the principle that systematic reviews on the effects of interventions will inform and help improve policy and services. Campbell offers editorial and methodological support to review authors throughout the process of producing a systematic review. A number of Campbell's editors, librarians, methodologists and external peer‐reviewers contribute.
	The Campbell Collaboration P.O. Box 7004 St. Olavs plass 0130 Oslo, Norway www.campbellcollaboration.org

### Note for authors

This document provides detailed methodological expectations for the conduct of Campbell Collaboration systematic reviews of *intervention effects*. It is important to note that some Campbell reviews may not focus on intervention effects, but may synthesise observational research that is policy relevant. For instance, such reviews may examine correlational or descriptive research, diagnostic or test accuracy, or other topics that do not necessarily focus on intervention effects. Although most of the methodological expectations listed below will be appropriate for all review topics (intervention focused or not), some (particularly those related to study design) may not be entirely applicable to nonintervention reviews, and have been noted as such under the “rationale and elaboration” column.

Status: Mandatory means that a new protocol or review will not be published if this standard is not met. Highly desirable means that this should generally be done but that there are justifiable exceptions. There may be legitimate variation between or within Campbell Coordinating Groups in the relative emphasis placed on compliance with highly desirable standards. The emphasis placed on compliance with highly desirable standards will remain at the discretion of each Campbell Coordinating Group. Optional means this is done at the authors' discretion.The Campbell Collaboration Policies and Guidelines document and the Cochrane Handbook (2019) may be helpful references for additional details on conduct standards.

**Table:**

Item No.	Status (T = Title, P = Protocol, R = Review)	Item Name	Standard	Rationale and elaboration	Authors note: pages where addressed
C1	Mandatory (T & P)	Formulating review questions	Ensure that the review question and particularly the outcomes of interest, address issues that are important to stakeholders such as consumers, practitioners, policy makers, and others	Campbell reviews are intended to support practice and policy, not just scientific curiosity. The needs of consumers play a central role in Campbell Reviews and they should play an important role in defining the review question	19‐20
C2	Mandatory (T & P)	Pre‐defining objectives	Define in advance the objectives of the review, including participants, interventions, comparators, and outcomes	Objectives give the review focus and must be clear before appropriate eligibility criteria can be developed. If the review will address multiple interventions, clarity is required on how these will be addressed (e.g., summarized separately, combined or explicitly compared).	19
C3	Highly desirable (P)	Considering potential adverse effects	Consider any important potential adverse effects of the intervention(s) and ensure that they are addressed	It is important that adverse effects are addressed if applicable in order to avoid one‐sided summaries of the evidence. In these cases, the review will need to highlight the extent to which potential adverse effects have been evaluated in any included studies. Sometimes data on adverse effects are best obtained from nonrandomized studies, or qualitative research studies. This does not mean however that all reviews must include nonrandomized studies	22
C4	Highly desirable (P)	Considering equity and specific populations	Consider in advance whether issues of equity and relevance of evidence to specific populations are important to the review, and plan for appropriate methods to address them if they are. Attention should be paid to the relevance of the review question to populations such as low socioeconomic groups, low or middle‐income regions, women, children, people with disabilities, and older people	Where possible reviews should include explicit descriptions of the effects of the interventions not only on the whole population but also describe their effects upon specific population subgroups and/or their ability to reduce inequalities and to promote their use to the community	21 and 22
C5	Mandatory (P)	Pre‐defining unambiguous criteria for participants	Define in advance the eligibility criteria for participants in the studies	Pre‐defined, unambiguous eligibility criteria are a fundamental pre‐requisite for a systematic review. The criteria for considering types of people included in studies in a review should be sufficiently broad to encompass the likely diversity of studies, but sufficiently narrow to ensure that a meaningful answer can be obtained when studies are considered in aggregate. Considerations when specifying participants include setting, age, identifying personal characteristics, demographic factors, and other factors that differentiate the participants. Any restrictions to study populations must be based on a sound rationale, since it is important that Campbell reviews are widely relevant	21
C6	Highly desirable (P)	Pre‐defining a strategy for studies with a subset of eligible participants	Define in advance how to handle studies in which only a subset of the sample is eligible for inclusion in the review	Sometimes a study includes some “eligible” participants and some “ineligible” participants, for example when an age cut‐off is used in the review's eligibility criteria. In case data from the eligible participants cannot be retrieved, a mechanism for dealing with this situation should be pre‐specified	21
C7	Mandatory (P)	Pre‐defining unambiguous criteria for interventions and comparators	Define in advance the eligible interventions and the interventions against which these can be compared in the included studies	Pre‐defined, unambiguous eligibility criteria are a fundamental pre‐requisite for a systematic review. Specification of comparator interventions requires particular clarity, including the extent to which the experimental interventions are compared with a control or comparison conditions with matched or similar participants. Any restrictions on interventions and comparators, such as regarding delivery, dose, duration, intensity, co‐interventions, and features of complex interventions should also be pre‐defined and explained	21‐22
C8	Mandatory (P & R)	Clarifying role of outcomes	Clarify in advance whether outcomes listed under “Criteria for Inclusion and Exclusion of Studies in the Review” are used as criteria for including studies (rather than as a list of the outcomes of interest within whichever studies are included)	Outcome measures need not always form part of the criteria for including studies in a review. However, some reviews do legitimately restrict eligibility to specific outcomes. For example, the same intervention may be studied in the same population for different purposes (e.g., reading interventions); or a review may address specifically the adverse effects of an intervention used for several conditions. If authors do exclude studies on the basis of outcomes, care should be taken to ascertain that relevant outcomes are not available because they have not been measured rather than simply not reported	22
C9	Mandatory (P)	Pre‐defining study designs	Define in advance the eligibility criteria for study designs in a clear and unambiguous way, with a focus on features of a study's design rather than design labels. For reviews with multiple objectives, specify whether study design inclusion criteria are common across all questions, or identified separately for each type of question	Pre‐defined, unambiguous eligibility criteria are a fundamental pre‐requisite for a systematic review. This is particularly important when nonrandomized (e.g., quasi‐experimental or observational) studies are considered. Some labels commonly used to define study designs can be ambiguous. For example a "double blind" study may not make it clear who is blind; a "case control" study may be nested within a cohort, or be undertaken in a cross‐ sectional manner; or a "prospective" study may have only some features defined or undertaken prospectively	20
C10	Mandatory (P, effectiveness reviews only)	Including randomized trials	Include randomized trials as eligible for inclusion in the review, if they are feasible and available for the interventions, outcomes, and populations of interest	Randomized trials are the best study design for evaluating the efficacy of many interventions. If they are feasible for evaluating questions that are being addressed by the review, they must be considered eligible for the review. However, appropriate exclusion criteria may be put in place, for example regarding length of follow‐up	20
C11	Mandatory (P)	Justifying choice of study designs	Justify the choice of eligible study designs	The particular study designs included should be justified with regard to appropriateness to the review question and with regard to potential for bias. It might be difficult to address some interventions or some outcomes in randomized trials. Authors should be able to justify why they have chosen either to restrict the review to randomized trials or to include nonrandomized studies	20‐21
C12	Mandatory (P & R)	Including studies regardless of publication status	Include studies irrespective of their publication status, and their electronic availability	Obtaining and including data from unpublished studies (including grey literature) can reduce the effects of publication bias.	21
C13	Mandatory (R)	Changing eligibility criteria	Justify any changes to eligibility criteria or outcomes studied. In particular, *post hoc* decisions about inclusion or exclusion of studies should keep faith with the objectives of the review rather than with arbitrary rules	Following pre specified eligibility criteria is a fundamental attribute of a systematic review. However unanticipated issues may arise. Review authors should make sensible *post hoc* decisions about exclusion of studies, and these should be documented in the review, possibly accompanied by sensitivity analyses. Changes to the protocol must not be based on findings of the studies or the synthesis, as this can introduce bias	‐
C14	Mandatory (P)	Pre‐defining outcomes	Define in advance which outcomes are primary outcomes and which are secondary outcomes	Pre‐definition of outcome reduces the risk of selective outcome reporting. The *primary outcomes* should be as few as possible (ideally no more than three). It is expected that the review should be able to synthesize these outcomes if eligible studies are identified, and that the conclusions of the review will be based in large part on the effect of the interventions on these outcomes	22
C15	Highly desirable (P)	Choosing outcomes	Keep the total number of outcomes selected for inclusion in the review as small as possible. Choose outcomes that are relevant to stakeholders such as consumers, practitioners, and policy makers. Consider the importance of resource use and cost outcomes	Campbell reviews are intended to support practice and policy, and should address outcomes that are important to consumers. These should be specified at protocol stage. Where they are available, established sets of core outcomes should be used. Participant‐reported outcomes should be included where possible. It is also important to judge whether evidence on resource use and costs might be an important component of decisions to adopt the intervention or alternative management strategies around the world. Large numbers of outcomes, while sometimes necessary, can make reviews unfocused, unmanageable for the user, and prone to selective outcome reporting bias	22
C16	Highly desirable (P)	Pre‐defining outcome details	Define in advance details of what are acceptable outcome measures (e.g., test scores conditions, characteristics, scales, composite outcomes)	Having decided what outcomes are of interest to the review, authors should clarify acceptable ways in which these outcomes can be measured	22
C17	Highly desirable (P)	Pre‐defining choices from multiple outcome measures	Define in advance how outcome measures will be selected at the coding stage when there are several possible measures (e.g., multiple definitions, assessors, or scales) or at the analysis stage if multiple effect sizes are coded per outcome construct	Pre‐specification guards against selective outcome reporting or selective analysis, and allows users to confirm that choices were not overly influenced by the results. A pre‐defined hierarchy of outcome measures may be helpful. It may however be difficult to pre‐ define outcome measures for adverse effects. A rationale should be provided for the choice of all outcome measures (including adverse effects)	22
C18	Highly desirable (P)	Pre‐defining time points of interest	Define in advance how differences in the timing of outcome measurement will be handled in the review	Pre‐specification guards against selective outcome reporting or selective analysis, and allows users to confirm that choices were not overly influenced by the results. Authors may consider whether all time frames or only selected time‐points will be included in the review. These decisions should be based on outcomes important for making policy or practice decisions. One strategy to make use of the available data could be to group time‐ points into pre‐specified intervals to represent “short‐term,” “medium‐term,” and “long‐ term” outcomes and to use information on no more than one from each interval from each study for any particular outcome	22‐23
C19	Mandatory (P)	Planning the search	Plan in advance the methods to be used for identifying studies. Design searches to capture as many studies as possible meeting the eligibility criteria, ensuring that relevant time periods and sources are covered and not restricting by language or publication status.	Searches should be motivated directly by the eligibility criteria for the review, and it is important that all types of eligible studies are considered when planning the search. There is a possibility of publication bias and/or language bias (whereby the language of publication is selected in a way that depends on the findings of the study) if searches are restricted by publication status or by language of publication. Removing language restrictions in English‐language databases is not a good substitute for searching non‐English language journals and databases.	23‐30
C20	Mandatory (P)	Planning the assessment of risk of bias/study quality in the included studies	Plan in advance the methods to be used for assessing risk of bias/study quality in included studies, including the tool(s) or codes to be used, how the tool(s) or codes will be implemented, and the criteria used to assign studies to risk of bias or quality categories (at outcome‐ and/or study‐level), for example, low risk, high risk, and unclear risk of bias; low quality or high quality	Pre‐defining the methods and criteria for assessing risk of bias/study quality is important because analysis or interpretation of the review findings may be affected by the judgments made during this process. For randomized trials, the Cochrane risk of bias tool is a recommended option	32
21	Mandatory (P)	Planning the synthesis of results	Plan in advance the methods to be used to synthesize the results of the included studies, including whether a quantitative synthesis is planned, how heterogeneity will be assessed, choice of effect measure (e.g., standardized mean difference, odds ratio, risk ratio), and methods for meta‐analysis (e.g., inverse variance or Mantel Haenszel, fixed‐effect or random‐ effects model). If a quantitative synthesis is not planned, or if it is not possible, plan the specific methods to narratively synthesize the results of the included studies	Pre‐defining the synthesis methods, particularly the statistical methods, is important because analysis or interpretation of the review findings may be affected by the judgments made during this process	34
C22	Mandatory (P)	Planning moderator analyses	Pre‐define potential effect modifiers for moderator analyses (e.g., subgroup analyses or meta‐regression analyses) at the protocol stage; restrict these in number; and provide rationale for each	Pre‐specification reduces the risk that large numbers of undirected moderator analyses lead to spurious explanations of heterogeneity	37
C23	Optional (P)	Planning a “Summary of findings” table	If a formal “Summary of findings” table is anticipated, specify which outcomes will be included, and which comparisons and subgroups will be covered (if appropriate)	The “Summary of findings table” offers a specific approach to summarizing the findings of a systematic review of intervention effects. Its use is not mandatory or recommended in Campbell Reviews of intervention effects but is highly desirable if the review is co‐registered with a Cochrane group. Methods for “Summary of findings” tables should be pre‐defined, particularly with regard to choice of outcomes, to guard against selective presentation of results in the review. If included, the table should include the essential outcomes for decision making (typically up to seven), which should generally not include surrogate or interim outcomes. These outcomes should not be chosen on the basis of any anticipated or observed magnitude of effect, or because they are likely to have been addressed in the studies to be reviewed. Outcome‐level summary risk of bias judgments made using the Cochrane Risk of Bias tool feed directly into the “Study limitations” column of a formal “Summary of findings table.” Therefore, authors planning a formal “Summary of findings table” should plan to use the Cochrane Risk of Bias tool in their assessments of risk of bias	NA
C24	Mandatory (P)	Planning the search	Refer to “Searching for Studies”, the Campbell information retrieval guide, to ensure that all relevant databases have been properly searched	Searches for studies should be as extensive as possible to reduce the risk of publication bias and to identify as much relevant evidence as possible. There is no minimum set of databases to search, but reviewers should consider consulting with a research retrieval specialist to avoid unnecessary duplication of effort	23‐30
C25	Highly desirable (P)	Searching specialist bibliographic databases	Search appropriate national, regional, and subject specific bibliographic databases	Searches for studies should be as extensive as possible to reduce the risk of publication bias and to identify as much relevant evidence as possible. Databases relevant to the review topic should be covered (e.g., ERIC for educational interventions, PsycINFO for psychological interventions), and regional databases (e.g., LILACS) should be considered	23
C26	Mandatory (if applicable) (P)	Searching for different types of evidence	If the review has specific eligibility criteria around study design to address adverse effects, economic issues, or qualitative research questions, undertake searches to address them	Sometimes different searches will be conducted for different types of evidence, such as for nonrandomized studies for addressing adverse effects, or for economic evaluation studies	25
C27	Mandatory (if applicable) (P)	Searching trials registers	When relevant, search trials registers and repositories of results, where relevant to the topic through ClinicalTrials.gov, metaREGISTER, the WHO International Clinical Trials Registry Platform (ICTRP) portal, and other sources as appropriate	When relevant, searches for studies should be as extensive as possible to reduce the risk of publication bias and to identify as much relevant evidence as possible. Although ClinicalTrials.gov is included as one of the registers within the WHO ICTRP portal, it is recommended that both ClinicalTrials.gov and the ICTRP portal are searched separately due to additional features in ClinicalTrials.gov	23‐24
C28	Mandatory (P)	Searching for grey literature	Search relevant grey literature sources such as reports/dissertations/theses databases and databases of conference abstracts	Searches for studies should be as extensive as possible to reduce the risk of publication bias and to identify as much relevant evidence as possible	23‐30
C29	Mandatory (P)	Searching within other reviews	Search within previous reviews on the same or similar topic	Searches for studies should be as extensive as possible to reduce the risk of publication bias and to identify as much relevant evidence as possible	29
C30	Mandatory (P)	Searching reference lists	Check reference lists in included studies and any relevant systematic reviews identified	Searches for studies should be as extensive as possible to reduce the risk of publication bias and to identify as much relevant evidence as possible	29
C31	Highly desirable (P)	Searching by contacting relevant individuals and organizations	Contact relevant individuals and organizations for information about unpublished or ongoing studies	Searches for studies should be as extensive as possible to reduce the risk of publication bias and to identify as much relevant evidence as possible. It is important to identify ongoing studies, so that when a review is later updated these can be assessed for possible inclusion	29
C32	Mandatory (R)	Structuring search strategies for bibliographic databases	Inform the structure of search strategies in bibliographic databases around the main concepts of the review, using appropriate elements from PICO and study design. In structuring the search, maximize sensitivity while striving for reasonable precision. Ensure correct use of the AND and OR operators	Inappropriate or inadequate search strategies may fail to identify records that are included in bibliographic databases. Expertise may need to be sought, in particular from an Information Retrieval Specialist. The structure of a search strategy should be based on the main concepts being examined in a review. In electronic bibliographic databases, a search strategy to identify studies for a Campbell Review will typically have three sets of terms: 1) terms to search for the population of interest; 2) terms to search for the intervention(s) evaluated; and 3) terms to search for the types of study designs to be included. There are exceptions, however. For instance, for reviews of complex interventions, it may be necessary to search only for the population or the intervention. Within each concept, terms are joined together with the Boolean “OR” operator, and the concepts are combined with the Boolean “AND” operator. The “NOT” operator should be avoided where possible to avoid the danger of inadvertently removing from the search set records that are relevant	‐
C33	Mandatory (R)	Developing search strategies for bibliographic databases	Identify appropriate controlled vocabulary (e.g., MeSH, Emtree, including “exploded” terms) and free‐text terms (considering, for example, spelling variants, synonyms, acronyms, truncation, and proximity operators), and tailor the search strategy to each specific database	Inappropriate or inadequate search strategies may fail to identify records that are included in bibliographic databases. Search strategies need to be customized for each database. It is important that MeSH terms are “exploded” wherever appropriate, in order not to miss relevant articles. The same principle applies to EMTREE when searching EMBASE and also to a number of other databases. The controlled vocabulary search terms are different for each electronic database, and thus search strategies must be tailored to each database. To be as comprehensive as possible, it is necessary to include a wide range of free‐text terms for each of the concepts selected. This might include the use of truncation and wildcards. Developing a search strategy is an iterative process in which the terms that are used are modified, based on what has already been retrieved	‐
C34	Highly desirable (R)	Using search filters	Use specially designed and tested search filters where appropriate (such as the Cochrane Highly Sensitive Search Strategies for identifying randomized trials in MEDLINE), but do not use filters in pre‐ filtered databases (e.g., do not use a randomized trial filter in CENTRAL or a systematic review filter in DARE or PROSPERO)	Search filters should be used with caution. They should be assessed not only for the reliability of their development and reported performance but also for their current accuracy, relevance, and effectiveness given the frequent interface and indexing changes affecting databases	‐
C35	Mandatory (P & R)	Restricting database searches	Justify the use of any restrictions in the search strategy on publication date, publication format, or language	Date restrictions in the search should only be used when there are date restrictions in the eligibility criteria for studies. They should be applied only if it is known that relevant studies could only have been reported during a specific time period, for example if the intervention was only available after a certain time point. Searches for updates to reviews might naturally be restricted by date of entry into the database (rather than date of publication) to avoid duplication of effort. Publication format restrictions (e.g., exclusion of letters) should generally not be used in Campbell reviews, since any information about an eligible study may be of value	23
C36	Mandatory (R)	Documenting the search process	Document the search process in enough detail to ensure that it can be reported correctly in the review/update. Include the month and year the search began and ended for future replicability	The search process (including the sources searched, when, by whom, and using what terms) needs to be documented in enough detail throughout the process to ensure that it can be reported correctly in the review, to the extent that all the searches of all the databases are reproducible	‐
C37	Highly desirable (R)	Rerunning searches	Rerun or update searches for all relevant databases within 12 months before publication of the review or review update, and screen the results for potentially eligible studies	The published review should be as up to date as possible. The search should be rerun close to publication, if the initial search date is more than 12 months (preferably 6 months) from the intended publication date, and the results screened for potentially eligible studies. Ideally the studies should be fully incorporated. If not, then the potentially eligible studies will need to be reported, at a minimum as a reference under “Studies awaiting classification” or “Ongoing studies”	‐
C38	Highly desirable (R)	Incorporating findings from rerun searches	Incorporate fully any studies identified in the rerun or update of the search within 12 months before publication of the review or review update	The published review should be as up to date as possible. After the rerun of the search, the decision whether to incorporate any new studies fully into the review will need to be balanced against the delay in publication	‐
C39	Highly desirable (P & R)	Making inclusion decisions in duplicate	The preferred procedure is for at least two members of the review team to independently screen candidate studies and resolve discrepancies by consensus. Where large numbers of studies are involved, samples of the candidate studies might be drawn and rescreened to estimate the reliability of the inclusion decisions	Duplicating the study selection process reduces both the risk of making mistakes and the possibility that selection is influenced by a single person's biases. The inclusion decisions should be based on the full texts of potentially eligible studies when possible, usually after an initial screen of titles and abstracts. It is desirable, *but not mandatory*, that two people undertake this initial screening, working independently	31
C40	Mandatory (P & R)	Including studies without useable data	Include studies in the review irrespective of whether measured outcome data are reported in a “usable” way	Systematic reviews typically should seek to include all relevant participants who have been included in eligible study designs of the relevant interventions and had the outcomes of interest measured. Reviews must not exclude studies solely on the basis of *reporting* of the outcome data, since this may introduce bias due to selective outcome reporting (i.e., that an effect size is not estimable although the outcome was clearly measured). While such studies cannot be included in meta‐ analyses, the implications of their omission should be considered. Note that studies may legitimately be excluded because outcomes were not *measured*. Furthermore, issues may be different for adverse effects outcomes, since the pool of studies may be much larger and it can be difficult to assess whether such outcomes were measured	33
C41	Mandatory (R)	Documenting decisions about records identified	Document the selection process in sufficient detail to complete a PRISMA flow chart and a table of “Characteristics of excluded studies”	A PRISMA flow chart and a table of “Characteristics of excluded studies” will need to be completed in the final review. Decisions should therefore be documented for all records identified by the search. Numbers of records are sufficient for exclusions based on initial screening of titles and abstracts. Broad categorizations are sufficient for records classed as potentially eligible during an initial screen. Studies listed in the table of “Characteristics of excluded studies” should be those which a user might reasonably expect to find in the review. At least one explicit reason for their exclusion must be documented. Authors will need to decide for each review when to map records to studies (if multiple records refer to one study). Lists of included and excluded studies must be based on studies rather than records	‐
C42	Mandatory (R)	Collating multiple reports	Collate multiple reports of the same study, so that each study rather than each report is the unit of interest in the review	It is wrong to treat multiple reports of the same study as if they are multiple studies. Secondary reports of a study should not be discarded, however, since they may contain valuable information about the design and conduct. Review authors must choose and justify which report to use as a source for study results	‐
C43	Mandatory (P & R)	Using data collection forms	Use a data collection form, which has been piloted	Review authors often have different backgrounds and level of systematic review experience. Using a data collection form ensures some consistency in the process of data extraction, and is helpful if comparing data extracted in duplicate. The original data collection forms should be included in the protocol for the review. If the data collection forms are altered during pilot testing, the final data collection forms should be submitted in an appendix with the final review	Supplementary material
C44	Mandatory (R)	Describing studies	Collect characteristics of the included studies in sufficient detail to populate final tables and narrative overview	Basic characteristics of each study will need to be presented as part of the review, including details of participants, interventions and comparators, outcomes and study design	‐
C45	Highly desirable (P & R)	Extracting study characteristics and outcome data in duplicate	The preferred procedure is for at least two members of the review team to independently code each study and resolve any discrepancies through discussion and consensus. Where large number of studies makes this procedure too demanding, random samples of the studies can be drawn and recoded by a different team member so that the reliability of the coding can be assessed and reported. The procedures planned for training coders and checking their accuracy before they begin providing data for the review should also be described along with the relevant background of those expected to do the coding	Duplicating the data extraction process reduces both the risk of making mistakes and the possibility that data selection is influenced by a single person's biases. Dual data extraction is particularly important for outcome data, which feed directly into syntheses of the evidence and hence to conclusions of the review	32
C46	Mandatory (P & R)	Making maximal use of data	Collect and utilize the most detailed numerical data that might facilitate similar analyses of included studies. Where 2×2 tables or means and standard deviations are not available, this might include effect estimates (e.g., odds ratios, regression coefficients), confidence intervals, test statistics (e.g., t, F, Z, chi‐squared), p‐values, or even data for individual participants	Data entry into most specialized computer software for meta‐analysis is easiest when 2×2 tables are reported for dichotomous outcomes or when means and standard deviations are presented for continuous outcomes. Sometimes these statistics are not reported but some manipulations of the reported data can be performed to obtain them. For instance, 2×2 tables can often be derived from sample sizes and percentages, while standard deviations can often be computed using confidence intervals or p‐values. Multiple software options are available for conversions.	32
C47	Highly desirable (R)	Examining errata	Examine any relevant retraction statements and errata for information	Some studies may have been found to be fraudulent or may for other reasons have been retracted since publication. Errata can reveal important limitations, or even fatal flaws, in included studies. All of these may potentially lead to the exclusion of a study from a review or meta‐analysis. Care should be taken to ensure that this information is retrieved in all database searches by downloading the appropriate fields together with the citation data	‐
C48	Highly desirable (P & R)	Obtaining unpublished data	Seek key unpublished information that is missing from reports of included studies	Contacting study authors to obtain or confirm data makes the review more complete, potentially enhancing precision and reducing the impact of reporting biases. Missing information includes details to inform risk of bias/study quality assessments, details of interventions and outcomes, and study results (including breakdowns of results by important subgroups)	34
C49	Mandatory (P & R)	Choosing intervention groups in multi‐arm studies	If a study is included with more than two intervention arms, include in the review only intervention and control groups that meet the eligibility criteria	There is no point including irrelevant intervention groups in the review. Authors should however make it clear in the “Table of characteristics of included studies” that these intervention groups were present in the study	34
C50	Mandatory (R)	Checking accuracy of numeric data in the review	Compare magnitude and direction of effects reported by studies with how they are presented in the review, taking account of legitimate differences	This is a reasonably straightforward way for authors to check a number of potential problems, including typographical errors in studies' reports, accuracy of data collection and manipulation, and data entry into a computer software program. For example, the direction of a standardized mean difference may accidentally be wrong in the review. A basic check is to ensure the same qualitative findings (e.g., direction of effect and statistical significance) between the data as presented in the review and the data as available from the original study. Results in forest plots should agree with data in the original report (point estimate and confidence interval) if the same effect measure and statistical model is used	‐
C51	Mandatory (P & R)	Assessing risk of bias/study quality	Assess the risk of bias/study quality for each included study, regardless of the study design or randomization type	Assessing risk of bias/study quality is an important task because it has been shown that risk of bias/study quality can influence estimates of intervention effects. If the review is co‐registered and uses randomized controlled trials, then the Cochrane Risk of Bias tool should be used. If not, then one of the many other study quality tools and/or coding schemes for study quality should be utilized and detailed within the protocol prior to implementation. Coding schemes for study quality are often used in addition to (or instead of) risk of bias/study quality tools in order to code specific quality variables relating to each source of bias/dimension of study quality. Campbell reviews should not use composite scales, indices, or other measures that conflate multiple measures of risk of bias/study quality into a single score (e.g., using an average scale that combines measures of allocation concealment, attrition, and baseline equivalence measures). These composite quality scales can be misleading and should not be used in a Campbell review. Instead, any risk of bias/study quality coding should isolate unique measures of quality (e.g., separate measures for allocation concealment, attrition, spillover, selective outcome reporting, selective analysis reporting, and baseline equivalence)	32
C52	Highly desirable (P & R)	Assessing risk of bias/study quality in duplicate	Use (at least) two people working independently to apply a risk of bias/study quality tool or coding scheme to each included study, and define in advance the process for resolving disagreements	Duplicating risk of bias/study quality assessment/coding reduces both the risk of making mistakes and the possibility that assessments are influenced by a single person's biases	33
C53	Highly desirable (R)	Supporting judgments of risk of bias/study quality	If applicable, justify categorical risk of bias/study quality judgments (e.g., high, low, and unclear) with information directly from the study	Providing support for the judgment makes the process transparent	‐
C54	Highly desirable (R)	Providing sources of information for risk of bias/study quality assessments	If applicable, collect the source of information for each risk of bias/study quality assessment. Where judgments are based on assumptions made on the basis of information provided outside publicly available documents, this should be stated	Readers/editors/referees should have the opportunity to see for themselves where supports for judgments have been obtained	‐
C55	Highly desirable (P & R)	Differentiating between performance bias and detection bias	Consider separately the risks of bias due to lack of blinding for (i) participants and study personnel (performance bias), and (ii) outcome assessment (detection bias)	The use of mutually exclusive domains of bias (e.g., selection bias, performance bias, detection bias, attrition bias and reporting bias) provides a more comprehensive framework for considering biases in randomized trials	32
C56	Only if applicable (R)	If applicable, assessing risk of bias due to lack of blinding for different outcomes	Consider blinding separately for different key outcomes.	The risk of bias due to lack of blinding may be different for different outcomes. When there are multiple outcomes, they should be grouped (e.g., objective versus subjective).	‐
C57	Only if applicable (R)	If applicable, assessing completeness of data for different outcomes	Consider the impact of missing data separately for different key outcomes to which an included study contributes data	When considering risk of bias due to incomplete (missing) outcome data, this often cannot reliably be done for the study as a whole. The risk of bias due to missing outcome data may be different for different outcomes. For example, there may be less drop‐out for a three‐month outcome than for a six‐year outcome. When there are multiple outcomes, they should be grouped (e.g., short term versus long term). Judgments should be attempted about which outcomes are thought to be at high or low risk of bias	‐
C58	Only if applicable (R)	If applicable, summarizing risk of bias assessments when using the Cochrane Risk of Bias tool	Summarize the risk of bias for each key outcome for each study	This reinforces the link between the characteristics of the study design and their possible impact on the results of the study, and is an important pre‐requisite for the GRADE approach to assessing the quality of the body of evidence	‐
C59	Highly desirable (R)	Addressing risk of bias/study quality in the synthesis	Address risk of bias/study quality in the synthesis (whether qualitative or quantitative). For example, present analyses stratified according to key risk of bias/study quality items, or conduct a moderator analysis with one or more risk of bias/study quality ratings	Review authors should consider how study biases affect conclusions. This is useful in determining the strength of conclusions and how future research should be designed and conducted	‐
C60	Highly desirable (R)	Incorporating assessments of risk of bias	If randomized trials have been assessed using one or more tools in addition to the Cochrane “Risk of bias” tool, use the Cochrane tool as the primary assessment of bias for interpreting results, choosing the primary analysis, and drawing conclusions	For consistency of approach across Campbell reviews, the Cochrane risk of bias tool should take precedence when two or more tools are used	‐
C61	Mandatory (R)	Combining different scales	If studies are combined with different scales, ensure that higher scores for continuous outcomes all have the same meaning for any particular outcome; explain the direction of interpretation; and report when directions were reversed	Sometimes scales have higher scores that reflect a “better” outcome and sometimes lower scores reflect “better” outcome. Meaningless (and misleading) results arise when effect estimates with opposite clinical meanings are combined	‐
C62	Mandatory (R)	Ensuring meta‐analyses are meaningful	Undertake (or display) a meta‐analysis only if participants, interventions, comparisons and outcomes are judged to be sufficiently similar to ensure an answer that is meaningful for the review question	A single mean effect size from a meta‐analysis of a very diverse collection of studies can be misleading. Variability in the nature of the treatment, control/comparison condition, sample characteristics, and intervention context, may be related to observed effects and a single mean effect size may misrepresent that diversity. Diversity does not necessarily indicate that a meta‐analysis should not be performed. However, authors must be clear about the underlying question that all studies are addressing and interpret the results appropriately. The determination of whether a meta‐analysis is meaningful should be made based on substantive knowledge of the effect sizes being synthesized; it should never be made based on statistical results for heterogeneity assessments	‐
C63	Mandatory (P & R)	Assessing statistical heterogeneity	Assess the presence and extent of between‐study variation when undertaking a meta‐analysis	The presence of heterogeneity affects the extent to which generalizable conclusions can be formed. It is important to identify heterogeneity in case there is sufficient information to explain it and offer new insights. Authors should recognize that there is much uncertainty in measures such as *I* ^2^ and *τ* ^2^ when there are few studies. Thus, use of simple thresholds to diagnose heterogeneity should be avoided	34
C64	Highly desirable (R)	Addressing missing outcome data	Consider the implications of missing outcome data from individual participants (due to losses to follow up or exclusions from analysis)	Incomplete outcome data can introduce bias. In most circumstances, authors should follow the principles of intention to treat analyses as far as possible (this may not be appropriate for adverse effects or if trying to demonstrate equivalence). Imputation methods can be considered (accompanied by, or in the form of, sensitivity analyses)	‐
C65	Highly desirable (R)	Addressing skewed data	Consider the possibility and implications of skewed data when analyzing continuous outcomes	Skewed data are sometimes not usefully summarized by means and standard deviations. While statistical methods are approximately valid for large sample sizes, skewed outcome data can lead to misleading results when studies are small	‐
C66	Mandatory (P & R)	Addressing studies with more than two groups	If multi‐arm studies are included, analyze multiple intervention groups in an appropriate way that avoids arbitrary omission of relevant groups and double‐ counting of participants.	Excluding relevant groups decreases precision and double counting increases precision spuriously; both are inappropriate and unnecessary. Alternative strategies include combining intervention groups, separating comparisons into different forest plots and using multiple treatments meta‐analysis	30
C67	Mandatory (P & R)	Comparing subgroups	If subgroup analyses are to be compared, and there are judged to be sufficient studies to do this meaningfully, use a formal statistical test to compare them	Concluding that there is a difference in effect across subgroups based on differences in the level of statistical significance within subgroups can be very misleading. Two groups may have similar treatment effects yet one may be statistically significant and the other not. Any conclusion that the intervention is effective for one group and not for the other should be based on a direct test of the mean difference between the groups (e.g., with meta‐analytic analog‐to‐the‐ANOVA or meta‐regression)	37
C68	Mandatory (P & R)	Interpreting subgroup analyses	If subgroup analyses are conducted, follow the subgroup analysis plan specified in the protocol without undue emphasis on particular findings. If post hoc subgroup analyses are conducted that were not specified in the protocol, the review must clearly state that these analyses are post hoc and exploratory in nature	Selective reporting, or over‐interpretation, of particular subgroups or particular subgroup analyses should be avoided. This is especially a problem when multiple subgroup analyses are performed. This does not preclude the use of sensible and honest post hoc subgroup analyses	37
C69	Mandatory (R)	Considering statistical heterogeneity when interpreting the results	Take into account any statistical heterogeneity when interpreting the results, particularly when there is variation in the direction of effect	The presence of heterogeneity affects the extent to which generalizable conclusions can be formed. If a fixed‐effect analysis is used, the confidence intervals ignore the extent of heterogeneity. If a random‐effects analysis is used, the result pertains to the mean effect across studies. In both cases, the implications of notable heterogeneity should be addressed. It may be possible to understand the reasons for the heterogeneity if there are sufficient studies	‐
C70	Mandatory (P & R)	Addressing nonstandard designs	Consider the impact on the analysis of clustering, matching, or other nonstandard design features of the included studies	Cluster‐randomized trials, cross‐over trials, studies involving measurements on multiple body parts, and other designs need to be addressed specifically, since a naive analysis might underestimate or overestimate the precision of the study. Failure to account for clustering is likely to overestimate the precision of the study—that is, to give it confidence intervals that are too narrow and a weight that is too large. Failure to account for correlation is likely to underestimate the precision of the study, i.e., to give it confidence intervals that are too wide and a weight that is too small	30
C71	Highly desirable (P & R)	Conducting sensitivity analysis	Use sensitivity analyses to assess the robustness of results, such as the impact of notable assumptions, imputed data, borderline decisions, and studies at high risk of bias or with poor quality	It is important to be aware when results are robust, since the strength of the conclusion may be strengthened or weakened	37
C72	Mandatory (R)	Interpreting results	Interpret a statistically nonsignificant p‐value (e.g., larger than 0.05) as a finding of uncertainty unless confidence intervals are sufficiently narrow to rule out an important magnitude of effect	Authors commonly mistake a lack of evidence of effect as evidence of a lack of effect	‐
C73	Highly desirable (R)	Investigating reporting biases	Consider the potential impact of reporting biases on the results of the review or the meta‐analyses it contains	There is overwhelming evidence of reporting biases of various types. These can be addressed at various points in the review. A thorough search, and attempts to obtain unpublished results, might minimize the risk. Analyses of the results of included studies, for example using funnel plots or regression tests for funnel plot asymmetry, can sometimes help determine the possible extent of the problem, as can attempts to identify study protocols, which should be a more routine feature of a review	‐
C74	Optional (P & R)	Including a “Summary of Findings” table	Include a “Summary of Findings” table according to recommendations described in Chapter 11 of the Cochrane Handbook (version 5 or later). Specifically:	For co‐registered reviews, a “Summary of Findings” table is highly desirable. For those reviews, authors should justify why a'Summary of Findings' table is not included if this is the case	NA
			□ include results for one population group		
			(with few exceptions);		
			□ indicate the intervention and the comparison intervention; □ include seven or fewer patient‐important outcomes; □ describe the outcomes (e.g., scale, scores, follow‐up); □ indicate the number of participants and studies for each outcome; □ present at least one baseline risk for each dichotomous outcome (e.g., study population or median/medium risk) and baseline scores for continuous outcomes (if appropriate); summarize the intervention effect (if appropriate); and □ include a measure of the quality of the body of evidence.		
C75	Optional (P & R)	Use the GRADE approach to assess the body of evidence	If the review is co‐registered with a Cochrane group, it is highly desirable to use the five GRADE considerations (study limitations, consistency of effect, imprecision, indirectness and publication bias) to assess the quality of the body of evidence for each outcome, and to draw conclusions about the quality of evidence within the text of the review. It is mandatory for all reviews to assess the quality of the body of evidence in some narrative or empirical manner; however, it is not mandatory that the GRADE approach be used to accomplish that goal	GRADE is the most widely used system for summarizing confidence in effects of the interventions by outcome across studies. It is preferable to use the GRADE tool (as implemented in GRADEprofiler and described in the help system of the software). This should help to ensure that author teams are accessing the same information to inform their judgments. If the GRADE tool is used, the five GRADE considerations should be addressed irrespective of whether the review includes a “Summary of Findings” table	NA
C76	Optional (R)	Justifying assessments of the quality of the body of evidence	Justify and document all assessments of the quality of the body of evidence (for example downgrading or upgrading if using the GRADE tool)	By adopting a structured approach, transparency is ensured in showing how interpretations have been formulated and the result is more informative to the reader	‐
C77	Mandatory (R)	Formulating implications for practice	Base conclusions only on findings from the synthesis (quantitative or narrative) of studies included in the review	The conclusions of the review should convey the essence of the synthesis of included studies, without selective reporting of particular findings on the basis of the result, and without drawing on data that were not systematically compiled and evaluated as part of the review	‐
C78	Highly desirable (R)	Avoiding recommendations	Avoid providing recommendations for practice	Campbell reviews should not attempt to tell people which interventions should or should not be used, since local considerations may be relevant. However, the implications of the findings should be discussed, and decision‐making can be helped by laying out different scenarios	‐
C79	Highly desirable (R)	Formulating implications for research	Structure the implications for research to address the nature of evidence required, including population intervention comparison, outcome, and type of study	Anyone wishing to conduct a study in the topic area of the review should be provided with a clear sense of what the remaining uncertainties are. A useful framework for considering implications for research is EPICOT (evidence, population, intervention, comparison, outcome and time stamp)	‐
